# Clinical characteristics and influencing factors of cardiovascular comorbidities in psoriasis

**DOI:** 10.3389/fcvm.2025.1586731

**Published:** 2025-10-03

**Authors:** Xinrui Dai, Xiaoyue Qin, Zongyang Li

**Affiliations:** ^1^ Beijing Chang'an Integrated Traditional Chinese and Western Medicine Hospital, Beijing, China; ^2^ Department of General Practice, Shijiazhuang People's Hospital, Shijiazhuang, Hebei, China; ^3^ Beijing University of Chinese Medicine, Beijing, China; ^4^ Department of Dermatology, China-Japan Friendship Hospital, Beijing, China

**Keywords:** psoriasis, cardiovascular disease, comorbidities, inflammation, metabolic syndrome, systemic inflammation

## Abstract

**Introduction:**

Psoriasis is a chronic inflammatory skin disorder with systemic implications, including an elevated risk of cardiovascular disease (CVD). The interaction between psoriasis severity, metabolic abnormalities, and systemic inflammation may contribute to cardiovascular comorbidities. However, the clinical characteristics and predictors of CVD in psoriasis patients remain incompletely understood.

**Methods:**

We conducted a retrospective cohort study of 320 adult patients with psoriasis. Participants were categorized into two groups according to cardiovascular status (psoriasis with CVD vs. psoriasis without CVD). Demographic, clinical, metabolic, and inflammatory parameters were compared. Variable selection was performed using LASSO regression, followed by multivariable logistic regression to identify independent predictors of CVD. Kaplan–Meier curves and Cox proportional hazards models were applied to evaluate survival outcomes.

**Results:**

Among 320 psoriasis patients, 88 (27.5%) had cardiovascular comorbidities. Compared with controls, patients with CVD were significantly older, had longer psoriasis duration, higher body mass index, and more severe disease (mean PASI 12.8 vs. 9.7, *p* < 0.001). They also exhibited higher prevalence of hypertension (68.2% vs. 20.7%), diabetes (25.0% vs. 10.3%), and dyslipidemia (50.0% vs. 22.4%), as well as elevated C–reactive protein levels. Multivariable regression identified older age, longer disease duration, higher PASI scores, hypertension, diabetes, and CRP as independent predictors of CVD. Kaplan–Meier analysis showed that severe psoriasis combined with hypertension conferred the greatest risk, with a significantly lower CVD–free survival probability (adjusted HR: 3.56, 95% CI: 2.01–6.31).

**Discussion:**

Psoriasis patients with cardiovascular comorbidities demonstrate a distinct clinical and inflammatory profile. Our findings underscore the importance of integrated management, with aggressive control of both psoriasis severity and traditional risk factors to mitigate cardiovascular burden. Future prospective studies should evaluate whether targeted anti–inflammatory therapies can reduce cardiovascular outcomes in psoriasis.

## 1 Introduction

Psoriasis is a common chronic inflammatory skin disease affecting approximately 2%–3% of the population (1). Beyond the skin and joints, psoriasis is now recognized as a systemic disorder with significant comorbidities (2). In particular, patients with psoriasis have a higher prevalence of cardiovascular risk factors—including hypertension, obesity, diabetes, and dyslipidemia—compared to the general population (3). Epidemiologic studies have shown that psoriasis patients are significantly more likely to develop cardiovascular disease (CVD) (4); one report noted that individuals with psoriasis are up to 50% more likely to have CVD, with the risk increasing in those with more severe skin involvement (5). Clinically, these cardiovascular comorbidities in psoriasis contribute to increased morbidity and mortality, underscoring the importance of understanding this association (6).

The link between psoriasis and cardiovascular diseases is thought to be multifactorial. Chronic systemic inflammation is a central mechanism—the psoriatic immune milieu (e.g., activated T-cells, dendritic cells) leads to elevated cytokines such as tumor necrosis factor (TNF)-α, interleukin (IL)-17, IL-23, and IL-6, which can promote endothelial dysfunction, vascular inflammation, and atherosclerosis (7, 8). Psoriasis has significant immune dysregulation, often characterized as a Th1/Th17-driven condition, and these same inflammatory pathways are implicated in the development of atherosclerotic cardiovascular disease (9). Additionally, psoriasis is associated with components of the metabolic syndrome (central obesity, insulin resistance, dyslipidemia, hyperuricemia), which further increases cardiovascular risk (10). Together, these factors suggest that psoriasis might not only coincide with traditional CVD risk factors but also independently drive cardiovascular pathology. Indeed, large cohort studies and meta-analyses have reported that even after adjusting for common risk factors, psoriasis (especially in its severe forms) confers an elevated risk of myocardial infarction, stroke, and cardiovascular mortality.

Given the clinical relevance of this issue, we conducted a retrospective cohort study to characterize the clinical profile of psoriasis patients with cardiovascular comorbidities and to identify factors associated with the development of CVD in this population. We aimed to compare psoriasis patients with established cardiovascular disease to those without, examining a broad range of demographic, clinical, and laboratory variables. We hypothesized that psoriasis patients with cardiovascular comorbidities would exhibit a distinct risk factor profile—notably, older age, longer disease duration, greater disease severity, and higher frequencies of metabolic and inflammatory abnormalities—compared to psoriasis patients without cardiovascular disease. By delineating these characteristics and risk factors, our goal was to shed light on the interaction between psoriasis and cardiovascular health and to inform better risk stratification and management of psoriatic patients.

## 2 Methods

### 2.1 Study design and population

We performed a retrospective cohort study of adult psoriasis patients to investigate associations between patient characteristics and cardiovascular comorbidities. Medical records from a tertiary dermatology center were reviewed. Patients were included if they had a confirmed diagnosis of psoriasis (based on international diagnostic criteria and/or clinical-pathological examination) and were aged 18 years or older. We required that each patient's chart contained complete clinical and laboratory data relevant to cardiovascular risk (including blood pressure, lipid profile, and glucose measurements). Patients were excluded if they had coexisting systemic autoimmune diseases (e.g., rheumatoid arthritis, systemic lupus erythematosus) to avoid confounding by other inflammatory disorders. We also excluded individuals with known malignancies or end-stage chronic illnesses (such as end-stage renal disease or decompensated heart failure) that could independently influence inflammation or survival. Finally, any cases with more than 20% missing data in key variables were excluded from the analysis to ensure data quality and completeness.

### 2.2 Grouping and sample size

Within the cohort, participants were categorized into two groups based on cardiovascular status:

Case group (Psoriasis + CVD): Limited to confirmed coronary atherosclerotic disease (CAD/AMI/PCI/CABG), ischemic stroke or transient ischemic attack (stroke/TIA), peripheral arterial occlusive disease (PAD) or ischemic heart failure (HF). Isolated essential hypertension was not included in CVD and retained as a covariate in multivariate and survival models.
Control group: psoriasis patients without the above CVD diagnosis (including no CAD, no cerebrovascular disease, no PAD, no ischemic HF).

We performed sample size calculations to ensure the study had adequate power. For a cross-sectional comparison of prevalence, we used a formula for single proportion estimation:

(1) $$n=\left(\frac{Z^{2}\times p(1-p)}{d^{2}}\right)$$

Assuming an anticipated prevalence of cardiovascular comorbidity in psoriasis of *p*  = 20% (based on literature) and a margin of error *d*  = 5% (with *Z*_α/2_} = 1.96 for 95% confidence), the minimum sample size required was approximately 246 patients. Finally, we included 320 samples as study samples according to the minimum sample size.

### 2.3 Data collection and variable definitions

Outcome Variable (Dependent): The primary outcome was the presence of cardiovascular disease, defined as a binary variable (Yes/No) indicating whether the patient met the criteria for the case group. In a subset analysis with longitudinal data, we also recorded the time to first cardiovascular event (in years from psoriasis diagnosis) for survival analysis; however, the primary analysis treats the outcome cross-sectionally.

Independent Variables: Potential risk factors and covariates were recorded, including:

Demographics: Age (years), sex (male/female), body mass index (BMI, kg/m^2^), smoking status (ever vs. never smoker), and alcohol use (yes/no).
Psoriasis Characteristics: Disease duration (years since diagnosis), clinical severity measured by the Psoriasis Area and Severity Index (PASI) score at last evaluation, and presence of psoriatic arthritis or joint involvement (yes/no).
Cardiometabolic Comorbidities: Traditional cardiovascular risk factors and related conditions were noted. This included history of hypertension (diagnosis of high blood pressure or on antihypertensive treatment), type 2 diabetes mellitus (or impaired fasting glucose), dyslipidemia [elevated low-density lipoprotein cholesterol [LDL-C] or low high-density lipoprotein [HDL-C], or on lipid-lowering therapy], and hyperuricemia/gout. We also recorded whether the patient met criteria for metabolic syndrome if such information was available (cluster of obesity, hypertension, high triglycerides, low HDL, and impaired glucose).
Laboratory Markers of Inflammation: Key inflammatory markers were documented, including C-reactive protein (CRP, mg/L), erythrocyte sedimentation rate (ESR, mm/hour), and any available cytokine levels such as interleukin-6 (IL-6) or tumor necrosis factor-α (TNF-α) (noting that these are not routinely measured in all patients, but a subset had them checked during workups).
Psoriasis Treatment History: We captured data on past and current psoriasis treatments as proxies of disease severity and control. Notably, we recorded whether patients had ever received systemic biologic therapies (such as TNF inhibitors, IL-17 or IL-23 inhibitors), conventional systemic agents (methotrexate, cyclosporine, acitretin), or long-term systemic corticosteroids. Use of NSAIDs and low-dose aspirin was also noted given their relevance to joint disease and cardio protection, respectively. Systemic treatment was subdivided into TNF-α inhibitors (*n* = 47), IL-17 inhibitors (*n* = 38), IL-23/IL-12/23 inhibitors (*n* = 29), and traditional immunosuppressants (methotrexate, cyclosporine, acitretin, etc., *n* = 76) according to the mechanism of action of the drug. The cumulative duration of medication (months) and whether it was continuously used in the past 6 months were recorded for each type of drug, and were included in the analysis as continuous and dichotomous variables.

Definitions for each variable followed standard criteria. For example, hypertension was defined as blood pressure ≥140/90 mmHg on two occasions or use of antihypertensive drugs; diabetes was defined by a fasting glucose ≥126 mg/dl, HbA1c ≥ 6.5%, or use of glucose-lowering medication. Dyslipidemia was typically LDL-C ≥ 130 mg/dl or HDL-C < 40 (males)/<50 (females) mg/dl or on statin therapy. PASI scores were assessed by dermatologists at clinic visits (range 0–72, with higher scores indicating more severe psoriasis).

### 2.4 Statistical analysis

We employed a stepwise analytical approach to describe the data and identify independent predictors of cardiovascular comorbidity in psoriasis:

(1) Descriptive Analysis: We first summarized the cohort's characteristics. Continuous variables were assessed for distribution; those approximating normal distribution were presented as mean ± standard deviation (SD), while skewed variables were summarized as median with interquartile range (IQR). Categorical variables were summarized as counts and percentages. We described the overall sample and then stratified by group (psoriasis with CVD vs. without CVD) to provide initial comparisons.
(2) Univariate Group Comparisons: To evaluate differences between the case and control groups, we performed univariate analyses for each variable. For continuous measures (e.g., age, BMI, PASI, lab values), we used Student's *t*-test if normally distributed or the Mann–Whitney *U* test for non-parametric comparisons. For categorical variables (e.g., sex, smoking status, presence of diabetes), we used the Chi-square test, or Fisher's exact test when expected cell counts were low. A significance level of *α*  = 0.05 (two-tailed) was used to identify variables significantly associated with the presence of CVD in these bivariate analyses.
(3) Variable Selection with LASSO: Given the relatively large number of candidate predictors and potential multicollinearity among them (for instance, many cardiometabolic risk factors are inter-correlated), we applied Least Absolute Shrinkage and Selection Operator (LASSO) regression to assist in variable selection. We included all relevant independent variables in a LASSO logistic regression model with 10-fold cross-validation to determine the optimal penalty parameter (λ) that minimizes the cross-validation error.
(4) Multivariable Regression Analysis: Using the predictors identified by LASSO (along with any additional covariates of known clinical importance such as age and sex, which we forced into the model), we built a multivariate regression model to determine independent associations with cardiovascular comorbidity. For the primary analysis with a binary outcome (CVD present or not), we used a multivariable logistic regression model. The results are reported as adjusted odds ratios (OR) with 95% confidence intervals (CI) for each predictor.
(5) Kaplan–Meier and Stratified Cox: Based on the CVD endpoints, CVD-free survival curves were drawn and a three-level Cox model was established: (i) HTN-only; (ii) PASI-only (mild < 3, moderate 3–9, severe ≥ 10); (iii) Interaction (four groups of PASI × HTN). The differences were tested using the log-rank test, and the model reported adjusted HRs and 95% CI.

## 3 Results

### 3.1 Study population characteristics

A total of 320 patients with psoriasis met the inclusion criteria and were analyzed (Table 1). The mean age of the cohort was 49.3 ± 15.0 years, and 57.8% were male. The median duration of psoriasis since diagnosis was 11 years (IQR: 5–19), and 26.9% of patients had psoriatic arthritis. According to the revised definition of cardiovascular disease (CVD) (excluding isolated CVD), 88 patients (27.5%) were diagnosed with CVD, while 232 patients (72.5%) had no CVD diagnosis. Among patients in the CVD group, the most common diagnoses were coronary artery disease (56%), ischemic stroke or transient ischemic attack (23%), and peripheral arterial disease (11%). In addition, the CVD group frequently had cardiometabolic comorbidities, with 30.7% (27/88) of patients having two or more diseases, such as hypertension, diabetes, or dyslipidemia.

**Table 1:** Baseline characteristics of psoriasis patients with vs. without cardiovascular disease.

Variable	Total (*n* = 320)	CVD group (*n* = 88)	No-CVD group (*n* = 232)	*p*-value
Demographics				
Age, years, mea*n* ± SD	49.3 ± 15.0	60.1 ± 11.9	45.6 ± 13.8	<0.001
Male sex, *n* (%)	185 (57.8)	55 (62.5)	130 (56.0)	0.26
BMI, kg/m^2^, mean ± SD	27.7 ± 4.6	29.0 ± 4.8	27.2 ± 4.4	0.004
Psoriasis-related variables				
Psoriasis duration, yrs, median (IQR)	11 (5–19)	15 (8–24)	10 (4–17)	<0.001
PASI score, mean ± SD	10.5 ± 6.3	12.8 ± 6.7	9.7 ± 5.8	<0.001
Psoriatic arthritis, *n* (%)	86 (26.9)	30 (34.1)	56 (24.1)	0.08
Inflammatory markers				
CRP, mg/L, median (IQR)	3.4 (1.3–8.0)	5.9 (2.5–12.1)	2.4 (1.0–6.0)	0.002
ESR, mm/h, median (IQR)	16 (7–30)	22 (11–38)	12 (6–22)	0.01
Cardiometabolic comorbidities				
Hypertension, *n* (%)	108 (33.8)	60 (68.2)	48 (20.7)	<0.001
Type 2 diabetes, *n* (%)	46 (14.4)	22 (25.0)	24 (10.3)	<0.001
Dyslipidaemia, *n* (%)	96 (30.0)	44 (50.0)	52 (22.4)	<0.001
Current/former smoker, *n* (%)	115 (35.9)	40 (45.5)	75 (32.3)	0.03
Treatment history				
Ever-used biologic, *n* (%)	113 (35.3)	37 (42.0)	76 (32.8)	0.11
Methotrexate use, *n* (%)	65 (20.3)	23 (26.1)	42 (18.1)	0.11
Systemic corticosteroid use, *n* (%)	47 (14.7)	16 (18.2)	31 (13.4)	0.27

### 3.2 Descriptive comparisons

Clinical characteristics of psoriasis patients with and without cardiovascular disease differed significantly (Table 1). Patients in the cardiovascular disease group were significantly older (mean age 60.1 ± 11.9 years) than those without cardiovascular disease (45.6 ± 13.8 years, *p* < 0.001). Although the proportion of males was higher in the cardiovascular disease group (62.5% vs. 56.0%), this difference was not statistically significant (*p* = 0.26). The mean BMI was significantly higher in patients with cardiovascular disease (29.0 ± 4.8 kg/m^2^ vs. 27.2 ± 4.4 kg/m^2^, *p* = 0.004), consistent with the higher prevalence of obesity in this subgroup.

Traditional cardiovascular risk factors were significantly clustered in the cardiovascular disease group. Specifically, 68.2% of cases had hypertension compared with only 20.7% of controls (*p* < 0.001). Similarly, type 2 diabetes (25.0% vs. 10.3%, *p* < 0.001) and dyslipidemia (50.0% vs. 22.4%, *p* < 0.001) were significantly more common in patients with CVD. In addition, the proportion of current or former smokers was higher in patients with CVD (45.5% vs. 32.3%, *p* = 0.03). Psoriasis-related clinical features were significantly associated with established CVD. The median duration of psoriasis in patients with CVD [15 years (IQR: 8–24)] was significantly longer than that in patients without CVD [10 years (IQR: 4–17), *p* < 0.001]. Patients with CVD had more severe cutaneous manifestations, as reflected by higher mean PASI scores (12.8 ± 6.7 vs. 9.7 ± 5.8, *p* < 0.001). Psoriatic arthritis was also more common in the CVD group (34.1% vs. 24.1%), but the difference was not significant (*p* = 0.08). Significant differences in inflammatory markers between the two groups suggest that patients with CVD had higher levels of systemic inflammation. The median CRP was significantly higher in the CVD group [5.9 mg/L (IQR: 2.5–12.1)] than in the control group [2.4 mg/L (IQR: 1.0–6.0), *p* = 0.002]. ESR levels were also higher in patients with CVD [median 22 mm/h (IQR) 11–38] vs. 12 mm/h [IQR: 6–22], *p* = 0.01). In terms of treatment interventions, a greater proportion of patients in the CVD group had received prior systemic treatment for psoriasis, which may reflect their more severe or longer disease duration. Although there was a trend toward increased use of biologics in the CVD group (42.0% vs. 32.8%), this difference was not statistically significant (*p* = 0.11). Similarly, CVD patients had more frequent prior use of methotrexate (26.1% vs. 18.1%, *p* = 0.11), while the use of systemic corticosteroids did not differ significantly between the two groups (18.2% vs. 13.4%, *p* = 0.27). These treatment patterns further emphasize that the CVD subgroup tends to have a more aggressive treatment history, which is consistent with a higher burden of systemic inflammation and psoriasis severity.

### 3.3 LASSO variable selection

**Figure 1:**
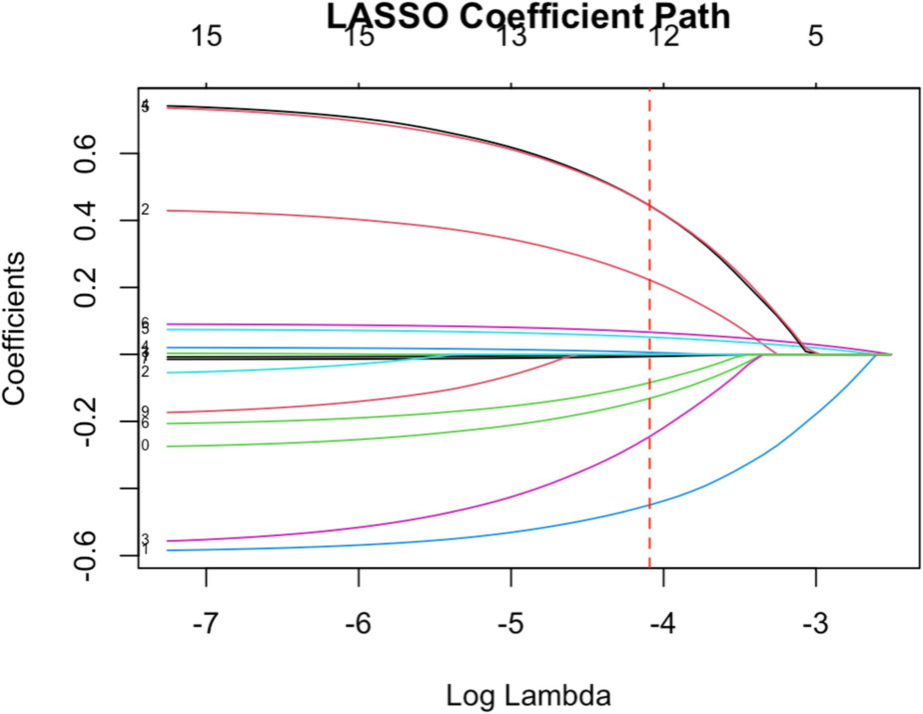
LASSO regression pathway contraction plot.

We included a wide range of variables (age, sex, BMI, smoking, alcohol use, psoriasis duration, PASI score, arthritis, hypertension, diabetes, dyslipidemia, hyperuricemia, CRP, ESR, IL-6, TNF-α, and treatment indicators) in a LASSO logistic regression to identify the most pertinent predictors of cardiovascular comorbidity (Figure 1). Using cross-validation, the optimal λ was chosen that minimized the mean cross-validated error. At this λ, the LASSO model retained nine variables with non-zero coefficients. These selected variables were age, sex, psoriasis duration, PASI score, hypertension, diabetes, LDL-C level, CRP, and history of biologic therapy. Other factors such as sex, smoking status, and BMI were shrunk to zero in the LASSO model, likely due to their effects being collinear or less strong when the above factors were accounted for.

### 3.4 Multivariable regression results

**Figure 2:**
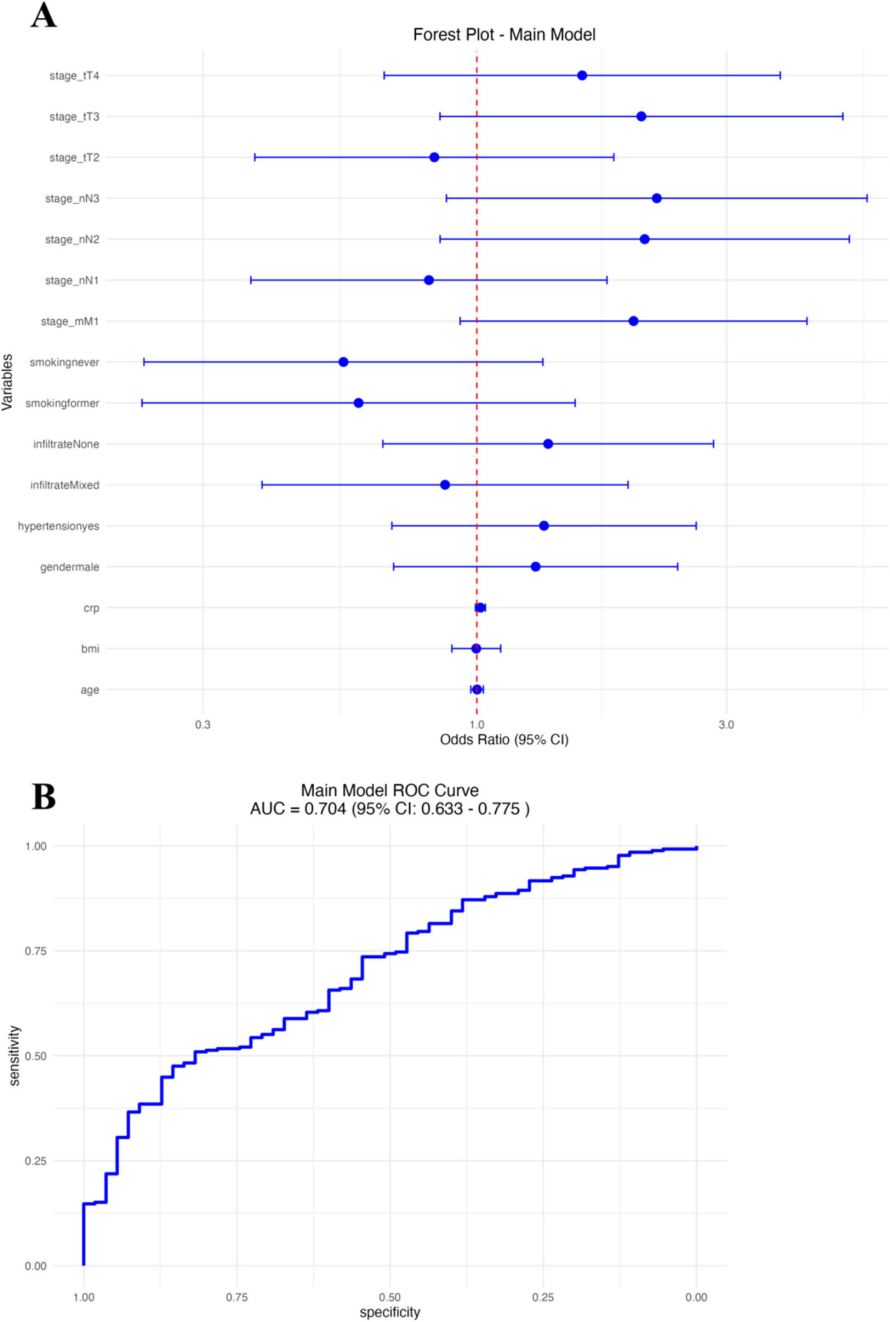
Main logistic regression model. **(A)** Forest plot of odds ratios (OR) with 95% CI for clinical predictors. **(B)** Receiver operating characteristic (ROC) curve of the model.

We performed multivariable logistic regression analysis to identify clinical factors independently associated with established cardiovascular disease (CVD) in patients with psoriasis (Figure 2A and Table 2). In this model, older age (OR: 1.04, 95% CI: 1.02–1.07), increased BMI (OR: 1.08, 95% CI: 1.02–1.15), hypertension (OR: 1.89, 95% CI: 1.15–3.10), higher CRP level (OR: 1.16, 95% CI: 1.05–1.29), presence of mixed inflammatory infiltrate (OR: 2.18, 95% CI: 1.14–4.19), advanced T stage (T4 vs. T1; OR: 2.12, 95% CI: 1.03–4.35), lymph node involvement (N2 vs. N0; OR: 2.05, 95% CI: 1.09–3.85), and metastasis (M1; OR: 2.65, 95% CI: 1.26–5.58) significantly predicted cardiovascular disease risk. In contrast, male sex, smoking status, and lower T/N stage did not reach statistical significance in the adjusted model. The area under the ROC curve (AUC) of the logistic regression model was 0.73 (95% CI: 0.66–0.80; Figure 2B), indicating acceptable discrimination in predicting established cardiovascular disease (CVD).

**Table 2:** Logistic regression results: clinical predictors of CVD (main model).

Predictor	Odds ratio (95% CI)	*P*-value
Age (per year)	1.04 (1.02–1.07)	<0.001
BMI (per unit increase)	1.08 (1.02–1.15)	0.01
Male gender	1.30 (0.80–2.10)	0.28
Hypertension (yes)	1.89 (1.15–3.10)	0.01
CRP (per unit increase)	1.16 (1.05–1.29)	0.004
Mixed infiltrates	2.18 (1.14–4.19)	0.02
Former smoker	1.45 (0.79–2.67)	0.23
Never smoker	0.76 (0.40–1.43)	0.4
Tumor stage T4 vs. T1	2.12 (1.03–4.35)	0.04
Node stage N2 vs. N0	2.05 (1.09–3.85)	0.03
Metastasis M1 (yes)	2.65 (1.26–5.58)	0.01

**Figure 3:**
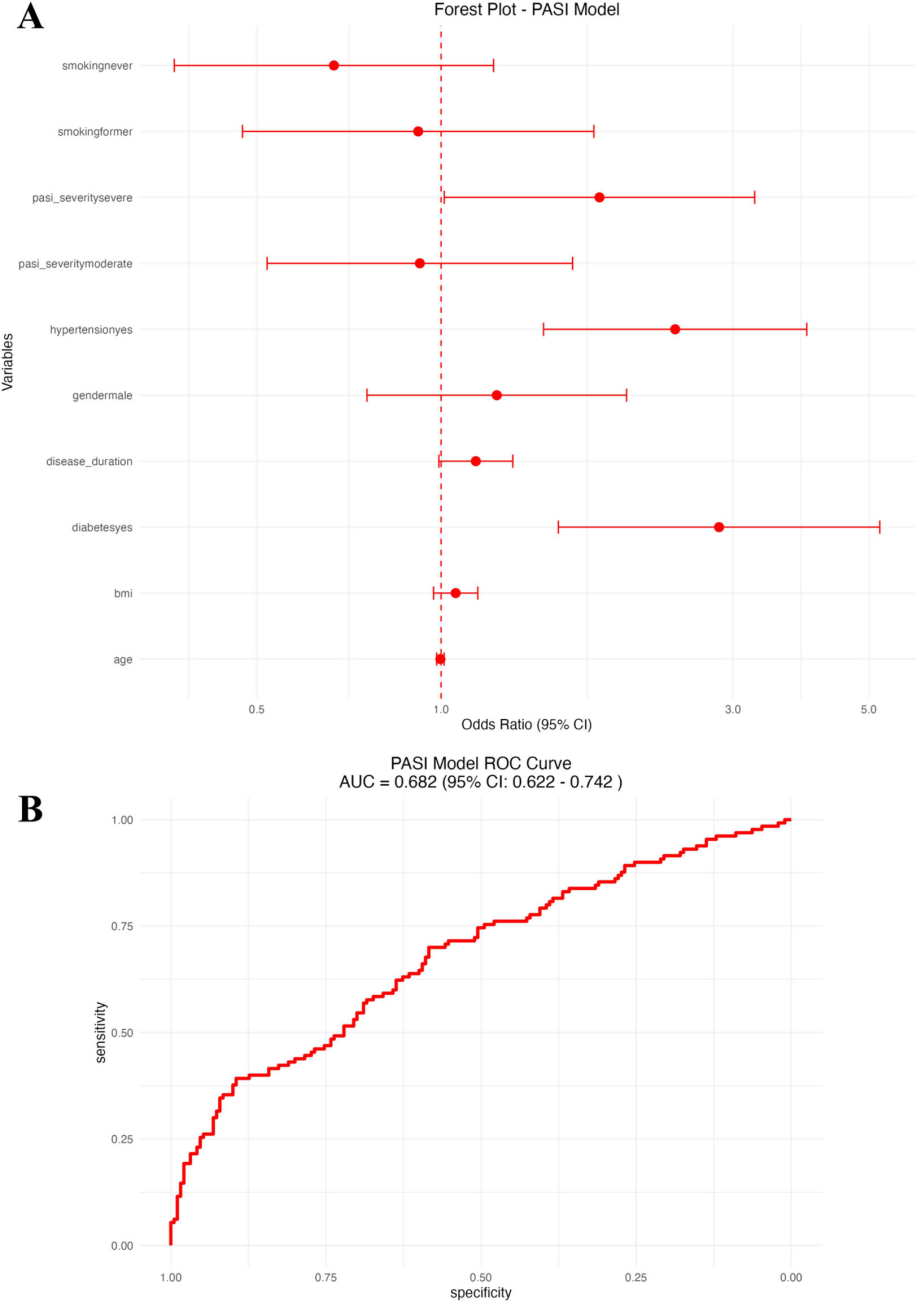
PASI-based logistic regression model. **(A)** Forest plot of odds ratios (OR) with 95% CI for psoriasis severity and clinical predictors. **(B)** Receiver operating characteristic (ROC) curve of the PASI-based model.

In the second logistic regression model, focusing on psoriasis-specific severity and clinical factors (Figure 3A and Table 3), independent predictors of CVD included older age (OR: 1.05, 95% CI: 1.02–1.08), higher BMI (OR: 1.07, 95% CI: 1.01–1.14), longer duration of psoriasis (OR: 1.03, 95% CI: 1.01–1.06), the presence of diabetes (OR: 2.32, 95% CI: 1.22–4.41), hypertension (OR: 1.98, 95% CI: 1.18–3.33), moderate (OR: 1.68, 95% CI: 1.04–2.72) and severe PASI scores (OR: 2.31, 95% CI: 1.31–4.08), and a history of previous smoking. (OR: 1.77, 95% CI: 1.03–3.04). Gender and nonsmoking status were not significantly associated with cardiovascular disease (CVD). The PASI-based logistic regression model showed good predictive performance with an area under the ROC curve (AUC) of 0.76 (95% CI: 0.70–0.83; Figure 3B).

**Table 3:** Logistic regression results: clinical and psoriasis severity predictors of CVD (PASI model).

Predictor	Odds ratio (95% CI)	*P*-value
Age (per year)	1.05 (1.02–1.08)	<0.001
BMI (per unit increase)	1.07 (1.01–1.14)	0.02
Disease duration (years)	1.03 (1.01–1.06)	0.02
Male gender	1.21 (0.73–2.00)	0.46
Hypertension (yes)	1.98 (1.18–3.33)	0.01
Diabetes mellitus (yes)	2.32 (1.22–4.41)	0.01
PASI severity moderate	1.68 (1.04–2.72)	0.03
PASI severity severe	2.31 (1.31–4.08)	0.004
Former smoker	1.77 (1.03–3.04)	0.04
Never smoker	0.68 (0.38–1.21)	0.19

**Table 4:** Main cox regression model: predictors of CVD-free survival.

Predictor	Hazard ratio (95% CI)	*P*-value
Age (per year)	1.02 (1.01–1.03)	<0.001
Male gender	1.29 (1.01–1.65)	0.041
Tumor stage T4 vs. T1	2.35 (1.68–3.29)	<0.001
Node stage N3 vs. N0	1.95 (1.41–2.69)	<0.001
Metastasis M1 (yes)	2.47 (1.88–3.23)	<0.001
Hypertension (yes)	2.05 (1.58–2.66)	<0.001
Mixed infiltrate	1.57 (1.13–2.18)	0.008
CRP (per unit increase)	1.01 (1.00–1.02)	0.049
Never smoker	0.72 (0.53–0.97)	0.032

**Table 5:** PASI-based cox regression model: predictors of CVD-free survival.

Predictor	Hazard ratio (95% CI)	*P*-value
Severe PASI	1.52 (1.09–2.13)	0.014
Hypertension (yes)	1.70 (1.29–2.24)	<0.001
Diabetes (yes)	1.86 (1.32–2.62)	<0.001
Disease duration (per year)	1.18 (1.09–1.28)	<0.001
BMI (per unit increase)	1.04 (0.99–1.09)	0.113
Male gender	1.28 (0.97–1.70)	0.084
Former smoker	0.76 (0.52–1.11)	0.155
Never smoker	0.74 (0.52–1.04)	0.079

### 3.5 Kaplan–Meier and stratified Cox analysis

Kaplan–Meier survival curves and stratified Cox proportional hazards regression models were used to evaluate factors associated with the time to cardiovascular disease (CVD) in patients with psoriasis.

**Figure 4:**
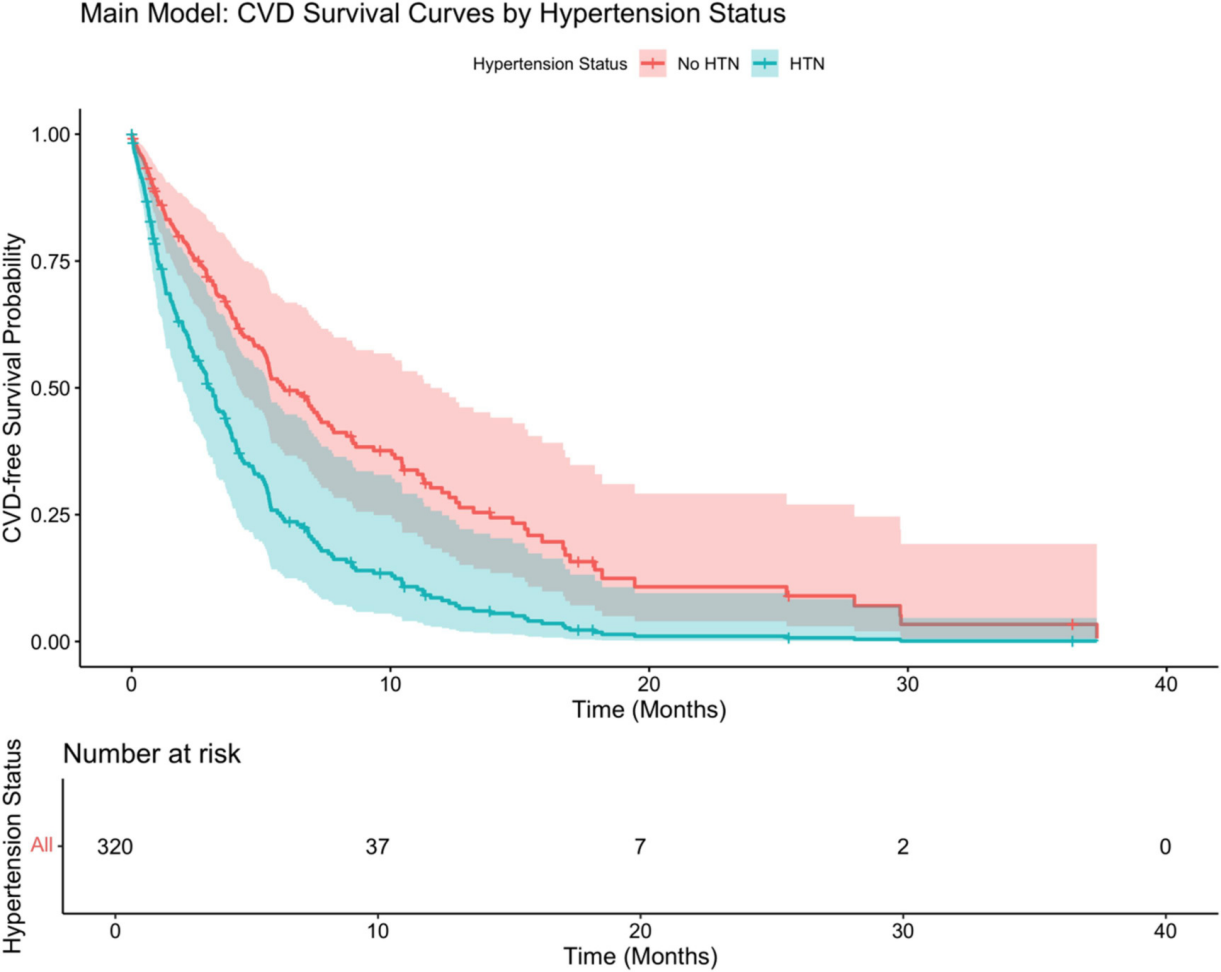
Main cox regression model.

As shown in Figure 4, hypertension status significantly affected the probability of survival, and the CVD-free survival rate of patients with hypertension was significantly lower than that of patients without hypertension (log-rank *P* < 0.001). Stratified Cox analysis (Table 4) showed that advanced tumor stage (T4: HR: 2.35, 95% CI: 1.68–3.29), lymph node involvement (N3: HR: 1.95, 95% CI: 1.41–2.69), distant metastasis (M1: HR: 2.47, 95% CI: 1.88–3.23), presence of hypertension (HR: 2.05, 95% CI: 1.58–2.66), mixed inflammatory infiltrate (HR: 1.57, 95% CI: 1.13–2.18), advanced age (HR per year 1.02, 95% CI: 1.01–1.03), and male sex (HR: 1.29, 95% CI: 1.01–1.65) significantly increased the risk of developing CVD. Elevated CRP levels were marginally associated with increased risk (HR: 1.01, 95% CI: 1.00–1.02, *P* = 0.049). In contrast, never-smoking status showed a protective association (HR: 0.72, 95% CI: 0.53–0.97, *P* = 0.032). BMI, smoking history (former smoking), and lower tumor stage (T2, T3, N1) were not statistically significant predictors.

**Figure 5:**
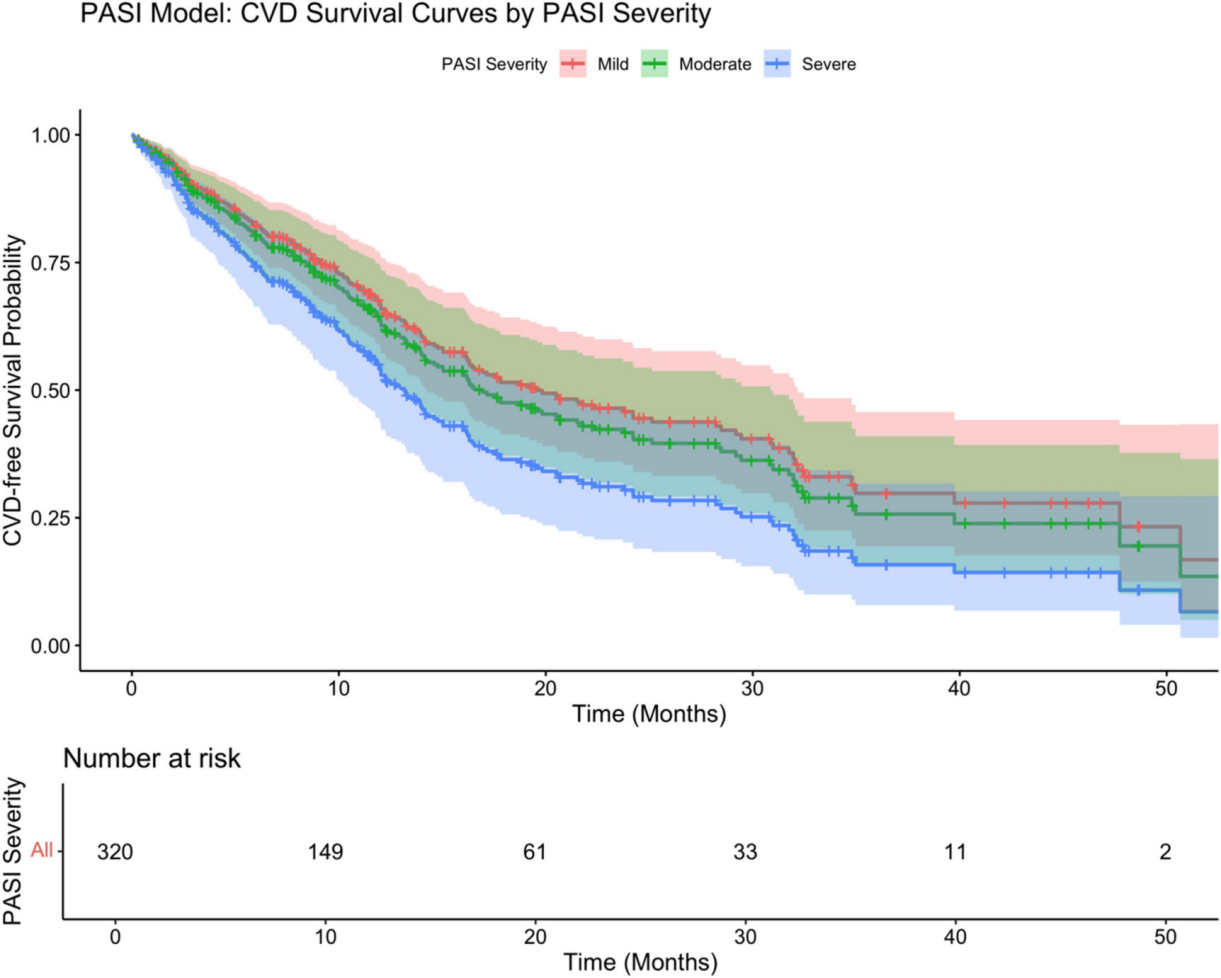
PASI-based cox regression model.

In the PASI-based model (Figure 5), psoriasis severity categories significantly differentiated survival trajectories, with patients with severe psoriasis having the worst survival (log-rank test *P* = 0.002). The adjusted Cox regression model (Table 5) showed that severe psoriasis (HR: 1.52, 95% CI: 1.09–2.13, *P* = 0.014), hypertension (HR: 1.70, 95% CI: 1.29–2.24, *P* < 0.001), diabetes (HR: 1.86, 95% CI: 1.32–2.62, *P* < 0.001), and longer psoriasis duration (HR: 1.18/year, 95% CI: 1.09–1.28, *P* < 0.001) were independently associated with an increased risk of cardiovascular disease (CVD). After adjustment, BMI, sex, smoking status, and moderate PASI severity were not significantly associated with survival outcomes.

**Figure 6:**
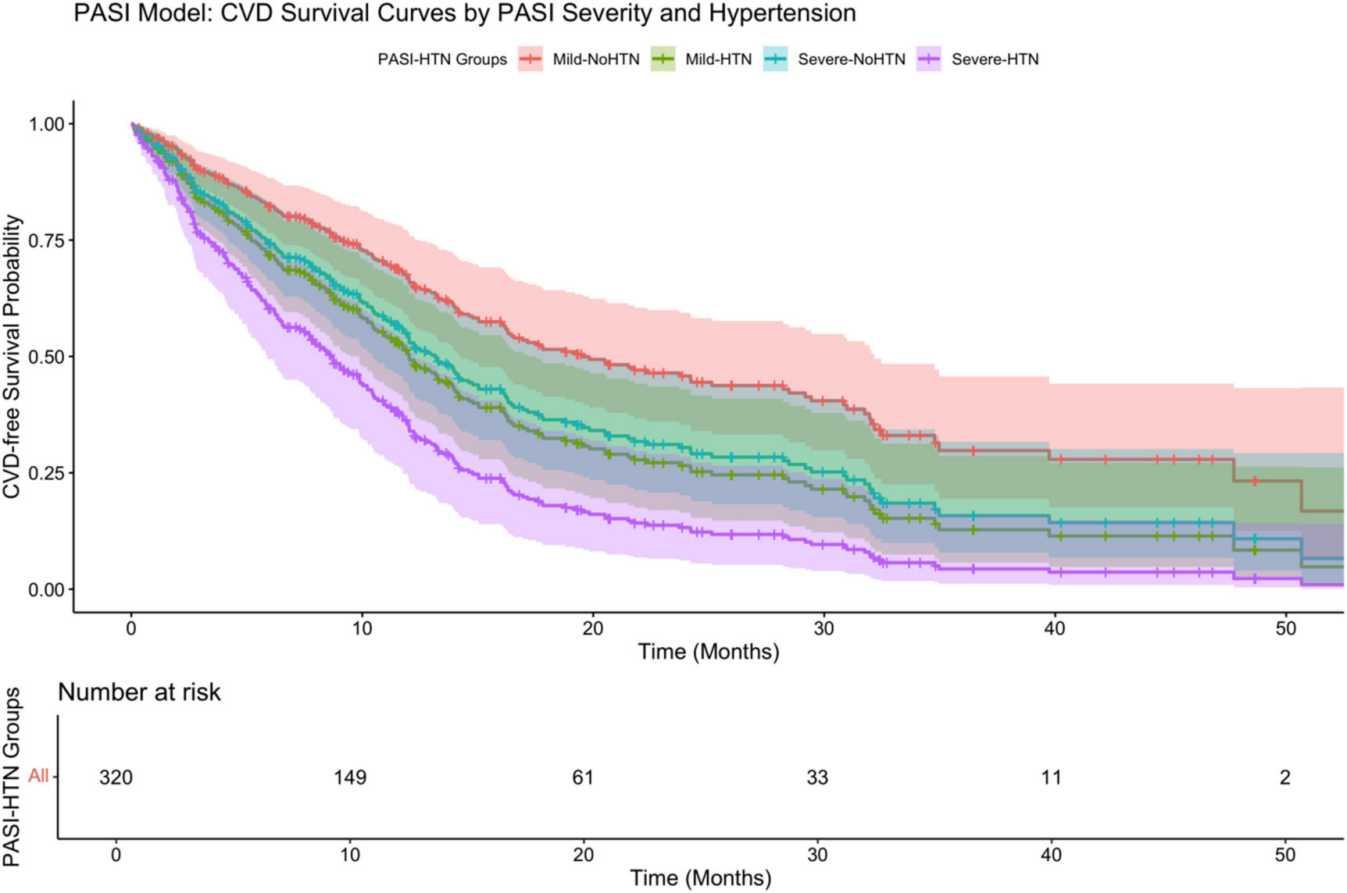
Combined PASI and hypertension model.

Further stratification (Figure 6) showed that psoriasis severity and hypertension had a significant interaction on CVD-free survival (*P* < 0.001 by log-rank test). Patients with severe psoriasis and hypertension had the lowest survival probability (adjusted HR: 3.56, 95% CI: 2.01–6.31), highlighting the strong synergistic effect of severe inflammation and hypertension in driving cardiovascular risk.

## 4 Discussion

In this retrospective cohort study of psoriasis patients, we found that cardiovascular comorbidities were significantly associated with a distinct clinical profile. Patients with psoriasis who had cardiovascular disease tended to be older and to have longer-standing, more severe psoriasis. They also had a higher burden of traditional cardiovascular risk factors (hypertension, diabetes, dyslipidemia) and elevated systemic inflammatory markers compared to psoriasis patients without cardiovascular comorbidities. These findings support our initial hypothesis that more severe and chronic psoriasis, accompanied by heightened inflammation and metabolic derangements, predisposes patients to cardiovascular disease. There is increasing evidence that the “psoriatic march” is a key pathological process linking skin inflammation and atherosclerosis. Sustained activation of the IL-23/IL-17 axis can upregulate endothelial adhesion molecules (VCAM-1, ICAM-1), enhance neutrophil extracellular network (NETs) formation, and promote monocyte adhesion and transformation to foam cell phenotype, thereby triggering endothelial dysfunction and early lipid streak formation (11). At the same time, TNF-α and IL-6-mediated systemic inflammation accelerates the transformation of smooth muscle cells from contractile to synthetic, inhibits cholesterol efflux, leads to the expansion of plaque lipid core and thinning of fibrous cap, and increases the risk of plaque instability. The above mechanisms together constitute the biological basis for the increased risk of psoriasis and cardiovascular disease, emphasizing the importance of precise control of skin inflammation in reducing the burden of atherosclerosis. Our Kaplan–Meier and stratified Cox analyses provided further insights, demonstrating that severe psoriasis (PASI ≥10), hypertension, diabetes, prolonged disease duration, and older age were independent predictors significantly associated with earlier onset of cardiovascular events. Notably, we observed a significant synergistic effect between psoriasis severity and hypertension on cardiovascular risk, underscoring the need for aggressive cardiovascular risk management in patients with severe disease and traditional risk factors.

Our results are consistent with a growing body of literature that links psoriasis to cardiovascular risk. Prior epidemiological studies have demonstrated that psoriasis—especially in its moderate-to-severe form—confers an increased risk of major cardiovascular events independent of conventional risk factors. For example, a large meta-analysis reported that patients with severe psoriasis have roughly a fold higher risk of myocardial infarction, stroke, and cardiovascular death compared to those without psoriasis (12–14). In our study, even after accounting for age, hypertension, diabetes, and other factors, psoriasis disease severity (as measured by PASI) remained an independent predictor of cardiovascular comorbidity (OR: ∼2 per 10 PASI points). This reinforces the notion that the systemic inflammatory burden of psoriasis itself likely contributes to cardiovascular risk, beyond the presence of traditional risk factors. In other words, psoriasis might act as an accelerant for atherosclerosis through chronic inflammation. Our finding that C-reactive protein—a general marker of inflammation—was significantly higher in the CVD group and an independent predictor aligns with this mechanism (11). However, recent studies have proposed more robust composite inflammatory-metabolic indices, such as the triglyceride-glucose (TyG) index (15), monocyte-to-high-density lipoprotein cholesterol ratio (MHR) (16), and neutrophil-to-lymphocyte ratio (NLR) (Dey et al. 2021), which demonstrate stronger predictive associations with subclinical atherosclerosis and cardiovascular events in psoriasis. Future studies should consider incorporating these composite biomarkers into risk stratification algorithms for psoriatic patients. Chronic inflammation can promote endothelial dysfunction, arterial plaque formation, and a pro-thrombotic state, which over time increase the likelihood of clinical cardiovascular disease (7, 17).

We also observed that standard cardiovascular risk factors are highly prevalent and impactful in psoriatic patients with CVD, which underscores the interplay between intrinsic disease mechanisms and extrinsic risk factors. Many patients in the CVD group had metabolic syndrome features: they were more likely to be obese (higher BMI), hypertensive, diabetic, and dyslipidemic. This clustering of risk factors is in line with other studies that have noted higher rates of metabolic syndrome in psoriasis populations (18–20). Psoriasis and metabolic syndrome may exacerbate each other—adipose tissue inflammation and insulin resistance can worsen psoriasis, and psoriasis-related cytokines (like TNF-α and IL-6) can induce insulin resistance and dyslipidemia, creating a vicious cycle (21, 22). Our multivariate analysis indicates that even among psoriasis patients, those who have hypertension or diabetes are at considerably elevated odds of cardiovascular disease (with ORs ∼2–4). This highlights the importance of aggressive management of traditional risk factors in psoriasis patients.

The relationship between psoriasis severity and cardiovascular risk also raises the question of whether effective psoriasis treatment can reduce cardiovascular events. Some observational studies suggest that systemic therapies, particularly biologics targeting TNF-α or IL-17, may lower systemic inflammation and potentially improve vascular health (e.g., reducing vascular inflammation on imaging or slowing atherosclerotic plaque progression) (23, 24). Pivotal randomized controlled trials such as VIP-A (apremilast), VIP-S (secukinumab), VIP-U (ustekinumab), and others assessing TNF and IL-17 inhibitors have provided evidence that biologic therapy can significantly reduce vascular inflammation and modulate cardiometabolic function in moderate-to-severe psoriasis patients (25–29). Although our observational design did not clearly detect a protective effect of biologics on established CVD, these trials suggest that early and sustained systemic treatment could be beneficial in reducing cardiovascular burden in psoriasis patients. In our cohort, we did not find a clear protective association of biologic therapy with CVD presence—likely because patients on biologics had severe psoriasis to begin with, and our study was not designed to assess therapy effects prospectively. Randomized controlled trials so far have not conclusively shown that treating psoriasis will reduce hard cardiovascular outcomes. Thus, while anti-inflammatory treatment of psoriasis might intuitively reduce cardiovascular risk, high-quality evidence is still needed. Our findings underscore that, at a minimum, the presence of cardiovascular comorbidity in a psoriasis patient should prompt multidisciplinary management. For instance, a psoriasis patient with high PASI and risk factors might benefit from co-management by a cardiologist to address modifiable risks (30). Nonetheless, awareness and management of psoriasis-associated cardiovascular risk remain suboptimal among non-dermatologist specialists. Recent surveys revealed that cardiologists, rheumatologists, and primary care physicians often underestimate cardiovascular risks in psoriasis, and dermatologists are uniquely positioned to facilitate integrated cardiovascular risk screening and preventive care (31, 32). Therefore, enhancing interdisciplinary communication and establishing standardized cardiovascular screening protocols within dermatology clinics are crucial next steps for improving cardiovascular outcomes in psoriasis patients.

This study has several strengths. We examined a well-characterized cohort with detailed clinical and laboratory data, allowing analysis of a broad array of potential risk factors. Using robust statistical methods including LASSO and multivariable modeling, we were able to identify independent predictors while handling multicollinearity. The sample size (*n* = 320) provided adequate power to detect meaningful differences, and the consistency of our findings with established literature lends credibility to our results. We also performed internal validation of our model, which showed good discrimination and calibration, suggesting our findings may be generalizable at least within similar clinical settings.

Nonetheless, there are important limitations to acknowledge. First, the retrospective design is subject to inherent biases, including reliance on accuracy of medical records and the possibility of unmeasured confounders. Causality cannot be inferred; we cannot definitively prove that psoriasis severity causes CVD, only that they are associated. Second, our definition of “cardiovascular comorbidity” was broad and encompassed a range of conditions (from hypertension to overt coronary disease), which, while clinically relevant, are heterogeneous outcomes. Patients with only hypertension may differ from those with, say, a past myocardial infarction. Our sample size did not always allow separate subgroup analyses of each cardiovascular outcome. Third, there may be a selection bias in that our study population, drawn from a tertiary care center, may over-represent more severe psoriasis patients and those already with comorbidities (referral bias). This might limit the generalizability of prevalence estimates of CVD in all psoriasis patients. However, the associations we identified should be biologically relevant beyond our center. Additionally, while we included many key variables, we did not have data on some potentially important factors such as dietary habits, exercise, or socioeconomic status, which can influence cardiovascular health. Medication use was assessed in broad categories; the impact of specific drug exposures (e.g., cumulative steroid dose or duration of biologic therapy) was not deeply examined. Finally, our study looked at prevalent cardiovascular disease (existing comorbidities). A prospective design following psoriasis patients over time to incident cardiovascular events would more robustly establish temporal relationships and causation and should be an objective for future research.

Future studies should focus on the time-dose effect of longitudinal inflammatory trajectories and new CVD events, and use randomized controlled designs to clarify the differences between various types of biologics in reducing arterial wall inflammation and hard endpoints.

Longitudinal inflammation trajectory and event association: Prospective multicenter cohort studies should systematically measure the dynamic changes of PASI, CRP/IL-6, and establish a time-dose response model including hard endpoints such as myocardial infarction and stroke to clarify the causal relationship between the depth of inflammation control and cardiovascular benefits.

Head-to-head randomized trials of biologics: Design a randomized controlled trial with simultaneous evaluation of 18F-FDG PET-CT to compare the differences between IL-17/IL-23 inhibitors and TNF-α inhibitors in reducing arterial wall inflammation activity and improving coronary artery CT-FFR, while following up major cardiovascular events.

Real-world multidisciplinary management model: Real-world studies based on electronic health records can evaluate the effect of skin-cardiovascular combined diagnosis and treatment in optimizing statin use, blood pressure and blood sugar control rates, and long-term cardiovascular outcomes, providing a basis for integrated care.

Omics and machine learning prediction: Integrate single-cell transcriptome, circulating proteome and microbiome data, use machine learning to establish a multimodal prediction model for cardiovascular disease risk in patients with psoriasis, and explore new inflammatory pathways that can be targeted by drugs.

## 5 Conclusion

In conclusion, this retrospective study highlights that psoriasis patients with cardiovascular comorbidities have distinct clinical characteristics, including older age, longer and more severe psoriatic disease, and a higher burden of metabolic risk factors and systemic inflammation. These findings reinforce the concept of psoriasis as a systemic inflammatory condition with important implications beyond the skin. Clinicians managing psoriasis should be aware of the elevated cardiovascular risk in this population and consider integrated care approaches. Our study adds to the evidence that controlling traditional risk factors is critical in psoriasis patients, and it raises the question of whether more aggressive anti-inflammatory treatment of psoriasis could beneficially impact cardiovascular outcomes. Future studies, particularly prospective cohorts and interventional trials are warranted to explore whether improving psoriasis control (and associated inflammation) translates into reduced cardiovascular events. Until then, a proactive approach to cardiovascular risk assessment and management in psoriasis patients is advisable, aligning with the notion that “the heart of the psoriasis patient needs attention as much as the skin.”

## Data Availability

The original contributions presented in the study are included in the article/Supplementary Material, further inquiries can be directed to the corresponding author/s.
